# Pyrrolidine dithiocarbamate activates the Nrf2 pathway in astrocytes

**DOI:** 10.1186/s12974-016-0515-9

**Published:** 2016-02-26

**Authors:** Jeffrey R. Liddell, Sarka Lehtonen, Clare Duncan, Velta Keksa-Goldsteine, Anna-Liisa Levonen, Gundars Goldsteins, Tarja Malm, Anthony R. White, Jari Koistinaho, Katja M. Kanninen

**Affiliations:** Department of Pathology, The University of Melbourne, Parkville, Victoria Australia; Mental Health Research Institute of Victoria, Parkville, Victoria Australia; Department of Neurobiology, A.I. Virtanen Institute for Molecular Sciences, University of Eastern Finland, Kuopio, Finland; Department of Biotechnology and Molecular Medicine, A.I. Virtanen Institute for Molecular Sciences, University of Eastern Finland, Kuopio, Finland

**Keywords:** Oxidative stress, Alzheimer’s disease, Copper, Metal

## Abstract

**Background:**

Endogenous defense against oxidative stress is controlled by nuclear factor erythroid 2-related factor 2 (Nrf2). The normal compensatory mechanisms to combat oxidative stress appear to be insufficient to protect against the prolonged exposure to reactive oxygen species during disease. Counterbalancing the effects of oxidative stress by up-regulation of Nrf2 signaling has been shown to be effective in various disease models where oxidative stress is implicated, including Alzheimer’s disease. Stimulation of Nrf2 signaling by small-molecule activators is an appealing strategy to up-regulate the endogenous defense mechanisms of cells.

**Methods:**

Here, we investigate Nrf2 induction by the metal chelator and known nuclear factor-κB inhibitor pyrrolidine dithiocarbamate (PDTC) in cultured astrocytes and neurons, and mouse brain. Nrf2 induction is further examined in cultures co-treated with PDTC and kinase inhibitors or amyloid-beta, and in Nrf2-deficient cultures.

**Results:**

We show that PDTC is a potent inducer of Nrf2 signaling specifically in astrocytes and demonstrate the critical role of Nrf2 in PDTC-mediated protection against oxidative stress. This induction appears to be regulated by both Keap1 and glycogen synthase kinase 3β. Furthermore, the presence of amyloid-beta magnifies PDTC-mediated induction of endogenous protective mechanisms, therefore suggesting that PDTC may be an effective Nrf2 inducer in the context of Alzheimer’s disease. Finally, we show that PDTC increases brain copper content and glial expression of heme oxygenase-1, and decreases lipid peroxidation in vivo, promoting a more antioxidative environment.

**Conclusions:**

PDTC activates Nrf2 and its antioxidative targets in astrocytes but not neurons. These effects may contribute to the neuroprotection observed for PDTC in models of Alzheimer’s disease.

**Electronic supplementary material:**

The online version of this article (doi:10.1186/s12974-016-0515-9) contains supplementary material, which is available to authorized users.

## Background

Oxidative stress results from an imbalance in the production and removal of reactive oxygen species (ROS) [1–3] and leads to induction of defensive gene expression that aims to detoxify ROS and promote cell survival [4]. An important transcription factor in the defense against oxidative stress is nuclear factor erythroid 2-related factor 2 (Nrf2). Nrf2 binds to the antioxidant response element (ARE) and induces the expression of ARE-driven cytoprotective genes, including heme oxygenase-1 (HO-1), NAD(P)H:quinone oxidoreductase-1 (NQO1), and glutamate-cysteine ligase (GCL), the rate-limiting enzyme of glutathione synthesis consisting of catalytic (glutamate-cysteine ligase catalytic subunit (GCLC)) and modifier (glutamate-cysteine ligase modifier subunit (GCLM)) subunits [5–7]. Nuclear Nrf2 abundance is regulated by positive and negative stimuli that affect nuclear import and export, binding to the ARE, and degradation [8, 9]. In the central nervous system, it appears that astrocytes, the most abundant glial cell type that possess important functions in the maintenance of brain homeostasis, have been implicated as the main target of Nrf2 activation [10] and increase their release of glutathione in response to Nrf2 activation [11].

During aging and disease, the normal compensatory mechanisms to combat ROS-mediated damage appear to be insufficient. Consistent with this, the level of nuclear Nrf2 is reduced in the hippocampus of Alzheimer’s disease (AD) brain [12], and attenuation of the Nrf2-ARE pathway coincides with disease progression in transgenic AD mice [13, 14], while genetic ablation of Nrf2 increases AD-associated pathology in AD mice [15]. Conversely, we have earlier shown that long-term overexpression of Nrf2 specifically in hippocampal neurons ameliorates memory deficits in a mouse model of AD [16] and transduction of primary neurons with Nrf2 protects against amyloid-beta (Aβ) toxicity [13].

In addition to overexpression, stimulation of the Nrf2-ARE pathway by small-molecule activators appears an appealing strategy to up-regulate the endogenous defense mechanisms of cells. At least nine classes of Nrf2-inducing agents have been described [17] with sulforaphane and tert-butylhydroquinone the most widely studied in the central nervous system [9, 18–20]. Some small-molecule activators have been used to assess their potential protective action in mouse models of AD [21–23], while dimethyl fumarate (Tecfidera®) has recently been approved for clinical use for relapsing form of multiple sclerosis [24].

Pyrrolidine dithiocarbamate (PDTC) is a blood-brain barrier permeant metal-chelating compound possessing antioxidant and anti-inflammatory properties. Many studies have reported induction of endogenous antioxidant gene expression and cytoprotective proteins by PDTC [25–29] and inhibition of nuclear factor-κB (NF-κB) [30–33]. The protective function of PDTC in animal models of central nervous system disorders has also been explored in several studies [34–40], while we and others have shown that PDTC is protective in AD mouse models [41, 42]. The protective mechanism of action of PDTC in AD involves modulation of brain copper concentration, glycogen synthase kinase 3β (GSK3β), and glial glutamate transporters, as well as suppression of NF-κB signaling. While we have recently shown that PDTC increases the proliferation of neural stem/progenitor cells in a manner similar to that caused by Nrf2 overexpression [43], the involvement of Nrf2 in PDTC-mediated effects remains unknown.

The present study aimed to explore the effect of PDTC on Nrf2 induction in normal conditions and in a setting of elevated Aβ. Here, we report that (i) Nrf2 is required for the PDTC-mediated enhancement of cellular resistance to oxidative damage, (ii) PDTC-induced Nrf2 activation occurs preferentially in astrocytes, and (iii) PDTC-mediated Nrf2 induction is amplified in the presence of Aβ.

## Methods

### Primary adult astrocyte culture

Cortices were isolated from 6- to 8-week-old C57Bl/6J mice and the Nrf2-disrupted C57BL/6JJcl mice generated by Itoh et al. [44] as described previously [45]. Following dissociation, the tissue was suspended in DMEM/F12 (3:1, Gibco) containing 10 % FBS and 100 U/mL penicillin-streptomycin (Gibco). The suspension was triturated and centrifuged at 1500 rpm for 5 min at room temperature. After adding 0.25 % trypsin-EDTA (Gibco), the suspension was incubated at 37 °C for 30 min. Fresh culture medium was added, and the suspension was centrifuged at 1500 rpm for 5 min. The cells were treated with 27 % Percoll (Sigma-Aldrich) in culture media, centrifuged at 1500 rpm for 10 min and washed once with fresh culture media. The cells were plated onto poly-l-lysine coated flasks in DMEM/F12 (3:1) containing 10 % FBS, 100 U/mL penicillin-streptomycin, and G5 supplement (Gibco). Cells were used between passages 3 and 7. These cultures are 99.8 % GFAP-positive, as determined by immunocytochemistry [45].

### Primary neonatal astrocyte culture

Primary astrocyte cultures were prepared from neonatal mice according to the method of Hamprecht and Löffler [46]. Briefly, as previously described [47], brains removed from newborn C57BL/6J mouse pups were mechanically dissociated by sequential passes through 250 and 135 μm nylon mesh, centrifuged at 500×*g* for 5 min before resuspension in DMEM supplemented with 50 U/mL penicillin-streptomycin and 10 % FBS and plated at 160,000 cells/cm^2^. The medium was renewed every 7 days, and cultures were used between 15 and 21 days in vitro, 1–2 days after medium renewal. Immunocytochemical analysis shows these cultures contain approximately 80 % GFAP-positive astrocytes with the remainder mainly IBA1-positive microglia but no neurons (data not shown).

### Primary hippocampal neuron culture

Hippocampi were dissected from embryonic day 18 mouse fetal brains and prepared as described previously [13]. Following dissociation, the cells were suspended in Neurobasal culture medium supplemented with 2 % B27, 500 μM glutamine, 25 μM glutamate, and 1 % penicillin-streptomycin (Gibco) and plated on 24- or 48-well culture plates precoated with poly-ornithine (0.5 μg/μL, Sigma-Aldrich) at 400,000 or 200,000 cells/well, respectively. One day after plating, the medium was supplemented with 10 μM cytosine β-D-arabinofuranoside and cells were grown in this media for 3 days prior to changing to media devoid of ﻿cytosine β-D-arabinofuranoside. Five days after plating, one third of the media was changed. Cells were treated at 7 days after harvesting. About 90 % of cells in these cultures are neurons [13].

### Primary mixed cortical culture

Primary mixed cultures of cortical neurons and astrocytes were prepared from embryonic day 14 mouse brains and maintained in neurobasal medium supplemented with 500 μM glutamine, 10 μg/mL gentamycin, and 2 % B27 as previously described [47]. Cells were used at 8 days in vitro, 2 days after renewal of medium. Neurons account for approximately 60–70 % of cells in these cultures, as determined by immunocytochemical staining for MAP-2 (data not shown).

### Cell treatments

PDTC (Sigma-Aldrich) was dissolved in sterile water and used immediately after preparation. The kinase inhibitors 10-DEBC, FPA-124, LY294002, PD198306, PD98059, PI828, SB415286, and SL327 (Tocris) were dissolved in DMSO to 10 mM. Aβ_(1-42)_ was purchased from American Peptide (Sunnyvale, CA) and dissolved to 1 mg/mL in sterile water. Protein concentration of the peptide was verified by DC protein assay kit (Bio Rad). The soluble peptide was used for experiments immediately after preparation as described [13].

### Viability assays

Cell viability following treatments was assessed by kinetically measuring the release of lactate dehydrogenase (LDH) into the culture medium using an LDH determination kit (Sigma-Aldrich/Roche). MTT reduction was assessed following incubation of cells with 480 μM MTT for 1 h before lysing in DMSO and quantifying absorbance at 560 nm. The data are expressed as percentage of control. For counting purposes, cells were fixed in 4 % paraformaldehyde for 30 min, stained with Hoechst, and enumerated from at least nine fields/treatment.

### Immunocytochemistry

After treatment, cells were fixed with 4 % paraformaldehyde for 20 min before blocking and permeabilizing in PBS containing 1 % BSA and 0.3 % triton X-100. Cells were subsequently incubated with antibody to Nrf2 (1:1000, Cell Signaling Technology (CST) #12721), MAP2 (1:1000; Chemicon, Merck Millipore), or GFAP (1:1000; Dako) overnight at 4 °C before further incubation with AlexaFluor-488, AlexaFluor-568, or AlexaFluor-647 secondary antibodies (1:250) for 1 h at room temperature. Before mounting onto microscope slides, cells were incubated with 0.5 μg/mL DAPI for 5 min. Cells were imaged with a Zeiss LSM Meta confocal microscope. Nuclear fluorescence intensity of Nrf2 was determined from approximately 500–700 cells per condition using ImageJ software.

### Nuclear fractionation

Following treatment, cells were washed twice with ice-cold PBS, collected into the PBS and centrifuged at 720×*g* for 3 min. Pellets were lysed in lysis buffer (50 mM Tris/HCl, 0.5 % triton X-100, 137.5 mM NaCl, 10 % glycerol, 1 mM sodium vanadate, 50 mM sodium fluoride, 10 mM sodium pyrophosphate, 5 mM EDTA, 1 % protease inhibitor cocktail (Sigma-Aldrich), pH 7.5) and resultant homogenates centrifuged at 15,000×*g* for 15 min. The supernatant was collected as cytosolic fraction before the pellet was rinsed with lysis buffer, re-centrifuged, and resuspended in lysis buffer containing 0.5 % SDS. Homogenates were sonicated before centrifuging at 15,000×*g* for 15 min and the supernatant collected as nuclear fraction. Samples were maintained at 4 °C throughout the procedure. Protein content was determined by BCA assay (Pierce).

### Gel electrophoresis and western blots

Proteins were harvested with PhosphoSafe buffer (Merck) containing protease inhibitor complete tablet (Complete, Roche Applied Sciences, Mannheim, Germany) or lysis buffer (as above). Proteins were separated on 12 % SDS-PAGE Tris-glycine gels. Proteins were transferred to PVDF membrane using Mini Trans-blot electrophoretic transfer cell equipment (Bio Rad) or Novex mini gels (Invitrogen) and then blocked in a solution of 5 % non-fat dry milk in 0.2 % Tween-20/0.01 M PBS for 30 min. For immunodetection of separated proteins, the membranes were incubated overnight at +4 °C with the following primary antibodies: HO-1 (Stressgen or CST), NQO1 (Abcam), Nrf2 (Santa Cruz Biotechnology or CST), Keap1 (CST), phospho-Akt (Ser473; CST), phospho-ERK1/2 (Thr202/Tyr204; CST), phospho-GSK3α/β (Ser21/9; CST), and β-actin (CST), GAPDH (CST), or histone H3 (CST) for loading controls. For detection, the membranes were incubated with HRP-conjugated Ig anti-rabbit, anti-mouse, or anti-goat secondary antibodies (1:5000; CST) for 2 h, developed using enhanced chemiluminescence (GE Healthcare ECL Advance Chemiluminescence kit) and imaged on a Fujifilm LAS3000 Imager (Berthold).

### Quantitative real-time PCR

RNA was isolated with RiboPure RNA kit (Life Technologies), and 500 ng of total RNA was used for cDNA synthesis using random hexamer primers (Promega, Madison, WI) and M-MuLV reverse transcriptase (New England Biolabs, Ipswich, MA) or Maxima reverse transcriptase (Thermo Scientific). The relative expression levels of mRNA encoding HO-1, GCLM, GCLC, NQO1, and Nrf2 were measured according to manufacturer’s protocol by quantitative RT-PCR (ABI PRISM 7700 Sequence detector, Applied Biosystems, Foster City, CA) using specific Assays-On-Demand target mixes (Applied Biosystems/Invitrogen). The expression levels were normalized to β-macroglobulin or β-actin and are presented as fold change in the expression over control.

### Glutamate-cysteine ligase activity assay

GCL activity was determined according to a modified method of White et al. [48]. Following treatment, cells were washed twice with ice-cold PBS before hypotonic lysis in 20 mM KP_i_ for 10 min on ice. Cells were quantitatively collected and centrifuged at 15,000×*g* for 10 min before 50 μL samples of supernatant were combined with 50 μL 400 mM Tris pH 7.4, 20 mM l-glutamic acid, 2 mM EDTA, 20 mM sodium borate, 2 mM l-serine, 40 mM MgCl_2_, and 40 mM ATP. For detection of basal glutathione and γ-glutamylcysteine content, duplicate 50 μL samples were combined with the same plus addition of 2 mM l-buthionine sulfoximine to inhibit GCL activity. Samples were warmed to 37 °C, and the GCL reaction was initiated by addition of 2 mM l-cysteine in 20 mM Tris pH 7.4, 1 mM EDTA, 250 mM sucrose, 20 mM sodium borate, and 2 mM l-serine. After incubation for 1 h at 37 °C, the GCL reaction was halted by addition of 50 μL ice-cold 200 mM sulfosalicylic acid. Aliquots (20 μL) were combined with reaction mix to final concentration of 1 mM 2,3-naphthalenedicarboxyaldehyde in 50 mM NaOH and 35 mM Tris for 30 min before fluorescence intensity was determined at 470ex/530em on an EnSpire multimode reader (PerkinElmer). Glutathione was used as standards. The difference between values in the absence and presence of l-buthionine sulfoximine is proportional to GCL activity, given as nmol γ-glutamylcysteine (γGC) generated per minute per milligram protein.

### Glutathione assay

Total glutathione content was determined according to the method of Tietze [49] as described previously [50]. For determination of cellular glutathione, 10 μL samples of cell lysates in 1 % sulfosalicylic acid were analyzed. For media analysis, one part medium was combined with one part water and two parts 1 % sulfosalicylic acid, and 20 μL of this was analyzed. Following addition of reaction mix to a final concentration of 200 μM NAPDH, 150 μM 5,5′-dithiobis-(2-nitrobenzoic acid), and 0.1 U glutathione reductase (Sigma-Aldrich) in 50 mM NaP_i_ buffer pH 7.5 containing 0.5 mM EDTA, the rate of thionitrobenzoate production was followed spectrophotometrically at 405 nm.

### Animal experiments

Starting from the age of 6 months, C57BL/6J wild-type (WT) mice were treated with PDTC (20 mg/kg/day) in drinking water for 9 months as previously described [41] (*n* = 6–8/group). A second cohort of mice was treated for 2 weeks at a dose of 75 mg/kg/day. The mice were deeply anesthetized with avertin and transcardially perfused with heparinized saline after which the brain was dissected and snap-frozen in liquid nitrogen. Brain tissue was also collected from Nrf2-deficient C57BL/6JJcl mice generated by Itoh et al. [44]. All animal work was approved by the National Animal Experiment Board of Finland (license number ESAVI-2011-000855) and followed the guidelines of the Council of Europe Legislation and Regulation for Animal Protection.

### Immunohistochemistry

Pentobarbital-anesthetized mice were transcardially perfused with heparinized saline. The brains were immersion-fixed with 4 % paraformaldehyde for 21 h and cryoprotected in 30 % sucrose for 48 h. The brains were frozen on liquid nitrogen and cut to 20-μm-thick cryosections. Sections were immunostained with HO-1 (Stressgen or CST) and GFAP (DakoCytomation, Glostrup, Denmark) overnight at room temperature. Primary antibody binding was detected by using Alexa Fluor 568-conjugated secondary antibody for HO-1 and Alexa Fluor 488-conjugated secondary antibody for GFAP (Invitrogen, Eugene, OR). The hippocampal dentate gyrus area from four to six sections in 200-μm intervals through the hippocampi were evaluated per animal. For quantification, the sections were imaged with an Olympus AX70 microscope (Olympus, Melville, NY) with an attached digital camera (Color View 12 or F-View; Soft Imaging System, Munster, Germany).

### Ferrous oxidation-xylenol orange (FOX) assay

Hippocampal tissues were sonicated in 90 % cold acetone solution and centrifuged at room temperature at 10,000×*g* for 10 min. Hydroperoxides were quantified from the supernatant based on detection of hydroperoxide-mediated oxidation of ferrous to ferric ion in the presence of xylenol orange [51] using hydrogen peroxide standards.

### Determination of brain copper content

The copper content of cerebella isolated from mice treated with PDTC for 2 weeks were measured by ICP-MS after freeze-drying and acid-digestion in nitric acid and hydrogen peroxide. The copper concentrations were determined with ICP-MS using external calibration and internal standard correction.

### Statistical analyses

The data were analyzed by using *t* test or ANOVA followed by Dunnett post hoc tests comparing to control condition in GraphPad Prism software, and statistical significance was assumed if *p* < 0.05. Unless otherwise mentioned, data are expressed as means ± SD.

## Results

### PDTC induces nuclear translocation of Nrf2 in astrocytes

Primary neurons and astrocytes were harvested from mouse brain to assess the ability of PDTC to induce the Nrf2 pathway. When used at 1- to 100-μM concentrations, PDTC did not cause astrocytic cell death as measured by the LDH release or MTT assay after 24 h treatment (Additional file 1: Figure S1A–B). PDTC did not cause death of hippocampal neurons when used at 1- to 50-μM concentrations (Additional file 1: Figure S1C).

**Fig. 1:**
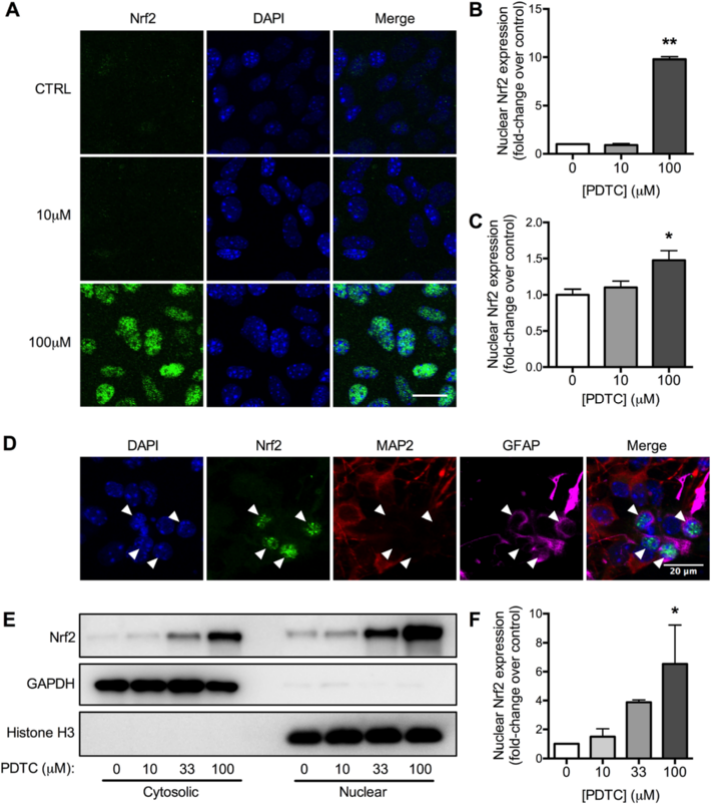
PDTC-induced translocation of astrocytic Nrf2. **a** Representative Nrf2 immunostaining images of primary neonatal astrocytes treated with 10 or 100 μM PDTC for 4 h. *Right panels* are merged images of Nrf2 (*left panels*) and the nuclear marker DAPI (*center panels*). **b** Quantification of nuclear Nrf2 immunostaining in astrocytes expressed as fold change over control ± SD. **c** Quantification of nuclear Nrf2 immunoreactivity in hippocampal neurons treated with PDTC for 4 h. Data are expressed as fold change over control cells ± SD. **d** Representative immunostaining of mixed cortical cultures treated with PDTC for 4 h, co-stained for DAPI, Nrf2, the neuronal marker MAP2 and astrocyte marker GFAP. *Arrowheads* indicate MAP2-negative GFAP-positive cells with strongly increased nuclear Nrf2. **e** A representative immunoblot with antibodies against Nrf2, GAPDH (cytosolic marker), and histone H3 (nuclear marker) with neonatal astrocyte samples treated with PDTC for 4 h. **f** Quantification of nuclear Nrf2 protein levels after 4 h PDTC treatment of astrocytes. Data are expressed as fold change over control cells normalized to histone H3 ± SD *p < 0.05; **p < 0.001. *Scale bar* = 20 μm

Nrf2 translocation into nuclei was first assessed by immunocytochemical staining of primary neonatal astrocytes treated with PDTC for 4 h (Fig. 1a). PDTC treatment induced a significant, tenfold increase in nuclear Nrf2 expression in astrocytes (Fig. 1b). A slight, 1.5-fold increase in nuclear Nrf2 was observed in hippocampal neurons treated with PDTC (Fig. 1c). Strong nuclear Nrf2 induction in mixed cortical cultures was restricted to MAP2-negative GFAP-positive cells (Fig. 1d). The specificity of the Nrf2 antibody and lack of PDTC effect in Nrf2-deficient cells was confirmed by absence of immunostaining in Nrf2^−/−^ astrocytes (Additional file 2: Figure S2). Nrf2 translocation was further confirmed by western blot of neonatal astrocytes after nuclear fractionation (Fig. 1e). PDTC induced a dose-dependent increase in the levels of nuclear Nrf2 in astrocytes (Fig. 1f).

### PDTC induces Nrf2 target genes in astrocytes but not neurons

**Fig. 2:**
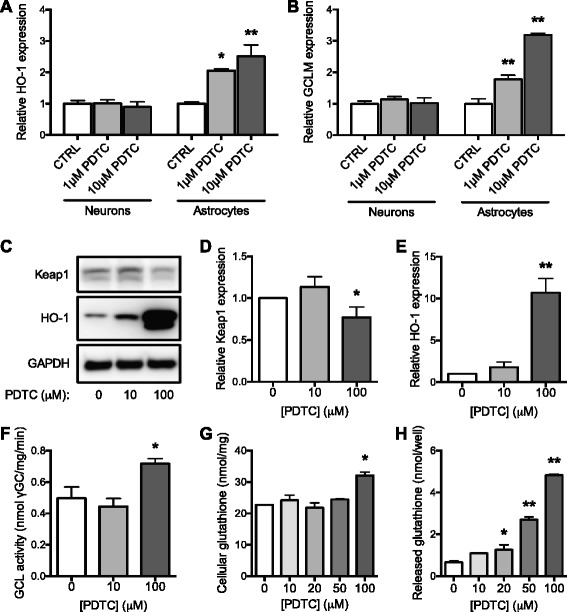
Effect of PDTC treatment on Nrf2 target genes, GCL activity, and glutathione content. **a** HO-1 and **b** GCLM mRNA expression levels measured by quantitative RT-PCR from primary adult astrocyte cultures and hippocampal neuron cultures after 24 h PDTC treatment. The mRNA expression levels are presented as mean relative expression normalized to β-actin ± SD. **c** Representative immunoblots with antibodies against Keap1, HO-1, and GAPDH from neonatal astrocytes treated with PDTC for 4 h. **d** Quantification of Keap1 and **e** HO-1 protein levels. Data are expressed as fold change over control cells normalized to GAPDH ± SD. **f** Neonatal astrocytes were treated with PDTC for 24 h, before GCL activity was determined. Data are expressed as mean nanomole γGC produced per minute per milligram protein ± SD. **g** The cellular glutathione content was measured after 24 h PDTC treatment of neonatal astrocytes and normalized to cellular protein content ± SD. Data are expressed as nanomole glutathione content per milligram protein. **h** Glutathione released into the media from neonatal astrocytes was measured 24 h after PDTC treatment. Data are shown as nanomole of glutathione released into the well ± SD. *p < 0.05; **p < 0.001 relative to corresponding control

Given that PDTC was a strong inducer of Nrf2 nuclear translocation, we next assessed the potential of PDTC to induce the expression of Nrf2 target genes. A 24-h treatment with PDTC induced a dose-dependent increase in Nrf2 target genes in adult astrocytes. HO-1 (Fig. 2a) and GCLM (Fig. 2b) mRNA expression levels were significantly elevated in astrocytes treated with 1 and 10 μM PDTC but not in hippocampal neurons. PDTC did not increase the mRNA expression levels of Nrf2, NQO1, or GCLC in either cell type (data not shown). Protein expression of HO-1 and the negative regulator of Nrf2, Keap1, was assessed by western blot in neonatal astrocytes treated with PDTC for 4 h (Fig. 2c). Keap1 expression decreased while HO-1 was strongly elevated (Fig. 2d, e).

### PDTC treatment increases GCL activity and glutathione levels in astrocytes

Because Nrf2 induction is known to increase glutathione biosynthesis in astrocytes, we investigated the potential of PDTC in this process. A 24-h PDTC treatment significantly increased GCL activity in neonatal astrocytes (Fig. 2f). Furthermore, cellular glutathione (GSH) levels and the release of GSH into the media were increased by PDTC (Fig. 2g, h), indicating that PDTC has potent antioxidant actions through GSH up-regulation.

### Nrf2 is more readily induced in adult astrocytes as compared to neonatal astrocytes

Significant up-regulation of HO-1 and GCLM occur with as little as 1 μM PDTC in adult astrocytes (Fig. 2a, b), whereas 100 μM PDTC is required to significantly increase HO-1 expression, GCL activity, and glutathione content in neonatal astrocytes (Fig. 2c, e–g). This indicates that astrocytes cultured from adult mice are more sensitive to PDTC than astrocytes cultured from neonatal mice.

### Nrf2 induction by PDTC involves kinase phosphorylation

**Fig. 3:**
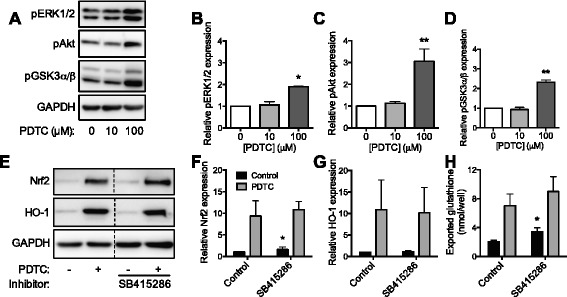
Involvement of kinases in Nrf2 induction by PDTC. **a** Representative immunoblots with antibodies against phosphorylated ERK1/2, phosphorylated Akt, phosphorylated GSK3α/β, and GAPDH in neonatal astrocytes treated with PDTC for 4 h. **b** Quantification of pERK1/2, **c** pAkt, and **d** pGSK3α/β protein levels. Data are expressed as fold change over control cells normalized to GAPDH ± SD. **e** Representative immunoblots with antibodies against Nrf2, HO-1, and GAPDH in neonatal astrocytes co-treated for 4 h with PDTC and SB415286 (GSK3 inhibitor). **f** Quantification of Nrf2 and **g** HO-1 protein expression after 4 h co-treatment of astrocytes with PDTC and SB415286. Data are expressed as fold change over control cells in absence of inhibitor, normalized to GAPDH ± SD. **h** Exported glutathione after 4 h PDTC co-treatment of neonatal astrocytes in presence of SB415286. Data expressed as nanomole glutathione released into the medium per well. *p < 0.05; **p < 0.01 relative to corresponding control

Increased phosphorylation of Akt and ERK1/2 and inhibitory phosphorylation of GSK3α/β were increased in PDTC-treated neonatal astrocytes (Fig. 3a–d), consistent with Nrf2 activation. The small-molecule GSK3 inhibitor SB415286 did not alter the PDTC-induced increase in Nrf2, HO-1, or GSH export but did significantly increase cellular Nrf2 levels and increase exported glutathione ~1.7-fold over cells treated in the absence of PDTC (Fig. 3e–h), suggesting the inhibitory phosphorylation of GSK3 induced by PDTC contributes to the activation of Nrf2. Cellular GSH levels were unchanged (data not shown). Inhibition of the ERK-activating mitogen-activated protein kinase kinase (MEK) by SL327, PD198306 or PD98059 did not significantly alter Nrf2 or HO-1 protein levels or GSH export (Additional file 3: Figure S3A–D), nor did inhibition of PI3K by LY294002 or PI828 and Akt by FPA124 or 10-DEBC (Additional file 3: Figure S3E–H). These inhibitors did not significantly decrease cellular GSH (data not shown) or significantly alter cellular viability with the exception of FPA124 and PI828, which mildly decreased viability (Additional file 4: Figure S4A-B).

### Nrf2 is required for the PDTC-mediated induction of NQO1 and up-regulation of glutathione biosynthesis

**Fig. 4:**
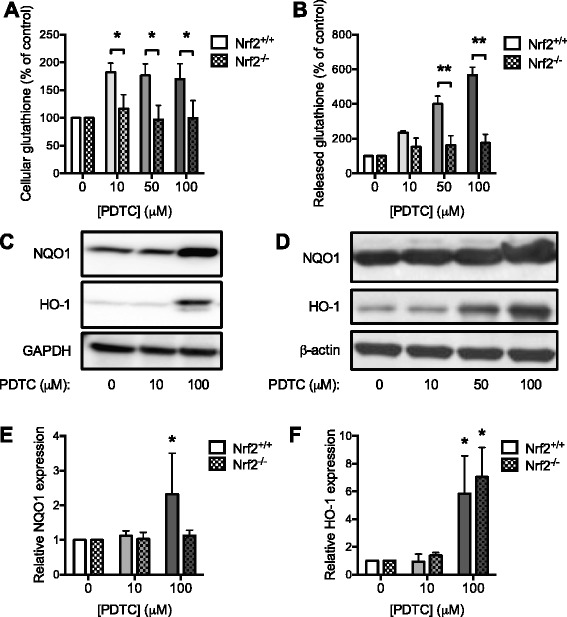
Absence of Nrf2 induction in Nrf2-deficient astrocytes. **a** Cellular and **b** released glutathione were determined from WT and Nrf2-deficient adult astrocytes treated with PDTC for 24 h. Data are expressed as percentage of control cells ± SD. **c** Representative immunoblots with antibodies against NQO1, HO-1, and GAPDH from WT astrocyte samples or **d** NQO1, HO-1, and β-actin from Nrf2^−/−^ astrocyte samples treated with PDTC for 24 h. **e** Quantification of NQO1 and **f** HO-1 protein expression in WT (Nrf2^+/+^) and Nrf2-deficient (Nrf2^−/−^) astrocytes treated with PDTC for 24 h. Data are expressed as fold change over control normalized to GAPDH or β-actin ± SD. *p < 0.05; **p < 0.001 relative to corresponding control

When compared to WT astrocytes, the PDTC-mediated increases in both cellular and released GSH were significantly reduced in Nrf2^−/−^ astrocytes (Fig. 4a, b), indicating that Nrf2 is central to the GSH-inducing effect of PDTC. Furthermore, PDTC induced astrocytic protein expression of HO-1 and NQO1 after 24 h treatment (Fig. 4c, e, f). Importantly, NQO1 expression was not induced by PDTC treatment in astrocytes harvested from Nrf2^−/−^ mice (Fig. 4d, e). Taken together, these results suggest that Nrf2 is required for PDTC-mediated induction of both glutathione biosynthesis and induction of protective protein expression.

### Co-treatment of PDTC with amyloid-beta magnifies Nrf2-ARE induction in astrocytes

**Fig. 5:**
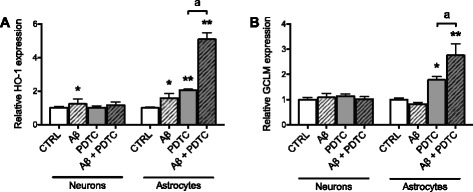
Effect of PDTC and Aβ co-treatment on Nrf2 target genes in primary astrocytes and neurons. **a** HO-1 and **b** GCLM mRNA expression levels were measured by quantitative RT-PCR from primary adult astrocyte cultures and hippocampal neuron cultures after 24 h treatment with 5 μM Aβ_(1-42)_ with or without co-treatment with 1 μM PDTC. The mRNA expression levels are presented as mean relative expression normalized to β-actin ± SD. *p < 0.05; **p < 0.001 relative to corresponding control; ^a^ *p* < 0.001 relative to PDTC condition

We have earlier shown that PDTC treatment [41] and Nrf2 overexpression [16] are protective in transgenic AD mice. To investigate the influence of an elevated Aβ-containing milieu on PDTC-mediated induction of the Nrf2-ARE, a co-treatment approach was applied. Treatment of hippocampal neurons and adult astrocytes with 5 μM Aβ_(1-42)_ alone caused a small increase in the expression of Nrf2-controlled HO-1, but not GCLM, after 24 h (Fig. 5a, b).

Co-treating cells for 24 h with Aβ and PDTC resulted in further substantial increases in astrocytic HO-1 (Fig. 5a), greater than that observed when treated with PDTC alone. HO-1 levels in neurons remained unchanged (Fig. 5a). While astrocytic NQO1 expression was not significantly increased by Aβ treatment (data not shown), a similar PDTC-mediated potentiation effect specifically in astrocytes was observed for GCLM mRNA expression levels (Fig. 5b), over that induced by PDTC alone.

Taken together, these results indicate that while the presence of Aβ slightly induces the expression of Nrf2 target genes, robust induction of the protective pathway is only achieved with co-treatment of PDTC and Aβ_(1-42)_, and this induction appears to be specifically restricted to astrocytes, not neurons.

### PDTC increases glial HO-1 expression and brain copper content in vivo

**Fig. 6:**
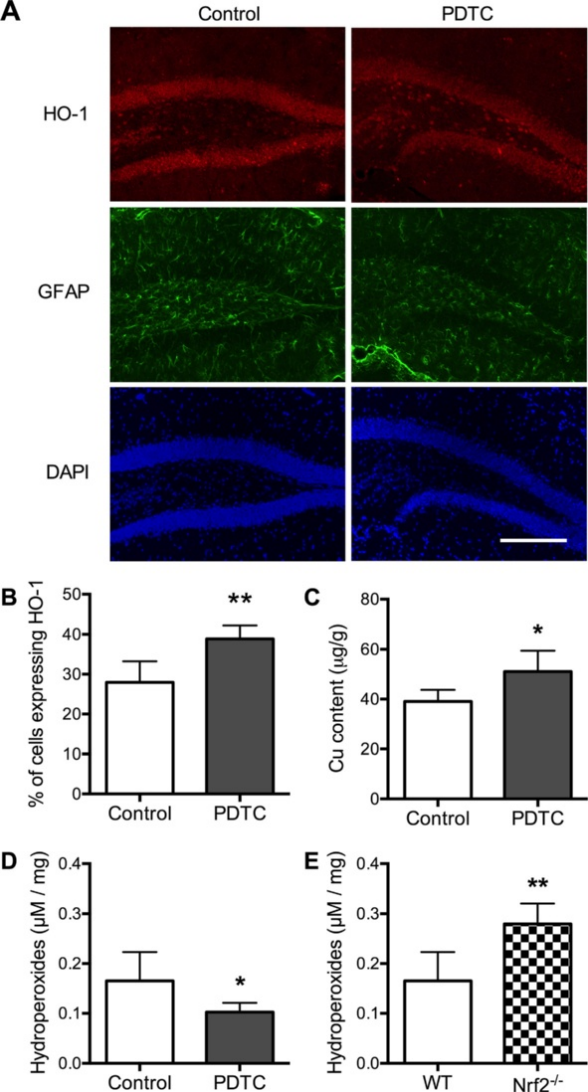
In vivo effects of PDTC on HO-1 expression, copper content and lipid peroxidation. **a** Representative immunostaining images of brain sections from WT mice treated with PDTC for 9 months with or without PDTC at a dose of 20 mg/kg/day. HO-1, GFAP, and nuclear DAPI were imaged from the hippocampal dentate gyrus. **b** The percentage of glial cells expressing HO-1 in the hippocampal dentate gyrus was calculated from five WT mice receiving PDTC for 9 months at a dose of 20 mg/kg/day and five vehicle-treated animals. Data are shown as percentage of HO-1 positive cells ± SD. **c** Cerebellar copper concentrations were measured from WT mice treated with PDTC for 2 weeks at a dose of 75 mg/kg/day by ICP-MS and are shown normalized to mass of the tissue ± SD. **d** Lipid peroxidation in hippocampi of WT mice treated for 9 months with or without PDTC at a dose of 20 mg/kg/day and in **e** WT and Nrf2^−/−^ hippocampi were measured by the FOX assay. Data are expressed as μM hydroperoxides per milligram protein ± SD. *p < 0.05; **p < 0.01

To assess the effect of PDTC treatment on expression of Nrf2 and target genes in the brain, we first measured mRNA expression levels of NQO1, HO-1, and Nrf2 and protein expression of Nrf2 and NQO1 in the hippocampi of WT mice treated with PDTC for 2 weeks at 75 mg/kg/day and in mice treated for 9 months at a dose of 20 mg/kg/day. PDTC treatment did not induce Nrf2-related genes (Additional file 5: Figure S5A–C) or protein expression of Nrf2 and NQO1 in the hippocampal tissue homogenates (Additional file 5: Figure S5D–F). To exclude the possibility that the lack of effect was due to PDTC not entering the brain, copper levels were assessed in the brains of PDTC-treated mice. As we have shown earlier [41], PDTC treatment caused a significant increase in copper levels in the cerebella (Fig. 6c), indicating that orally administered PDTC penetrated the brain but failed to induce Nrf2-controled gene and protein expression when assessed from tissue homogenates.

To assess the in vivo effect of oral PDTC administration in more depth and to determine the cell-specific effects of PDTC, we next quantified the protein expression of HO-1 in the hippocampal dentate gyrus of mice treated with PDTC for 9 months at a dose of 20 mg/kg/day. The expression of glial HO-1 was increased in PDTC-treated animals in the hippocampal dentate gyrus (Fig. 6a, b). This suggests that in contrast to the lack of PDTC effect in whole tissue homogenates, significant induction of the Nrf2 target gene HO-1 can be achieved in specific cell populations.

### PDTC treatment enhances resistance to lipid peroxidation, which is increased in Nrf2 deficiency in vivo

To assess the potential of PDTC in reducing lipid peroxidation, 6-month-old WT mice were treated orally with PDTC for 9 months at a dose of 20 mg/kg/day according to [41]. As assessed by the FOX assay, oral PDTC treatment reduced the amount of hippocampal hydroperoxides by 62 % (Fig. 6d), thus providing further evidence that PDTC enhances cellular resistance to oxidative damage. Hydroperoxides were increased by 47 % in the Nrf2^−/−^ hippocampi when compared to WT controls (Fig. 6e).

## Discussion

The crucial function of the Nrf2 pathway in central nervous system disorders is demonstrated by in vivo studies where Nrf2 induction is associated with improvements in behavior and functional outcomes, and lack of Nrf2 is associated with worsened outcome or reduced neuroprotection [52–55], including AD [15]. Correspondingly, Nrf2 appears to be suppressed in the hippocampi of AD patients [12]. Hence, Nrf2 is emerging as an important potential therapeutic target. In recent years, a variety of small-molecule activators of the Nrf2-pathway have been described. These represent an important pharmacological means to control Nrf2 activation. The present study identifies PDTC as a novel inducer of the Nrf2 pathway specifically in astrocytes. This study also finds that further augmentation of Nrf2 induction by PDTC occurs in the presence of Aβ and demonstrates the critical requirement of Nrf2 for the PDTC-mediated enhancement of cellular resistance to oxidative damage.

While we have earlier shown that induction of neuronal Nrf2 is protective in AD models [13, 16], it is generally considered that Nrf2 induction occurs primarily in astrocytes and that this induction confers protection upon neurons [10, 56–59]. For example, Nrf2-mediated glutathione release from astrocytes protects neurons [59]. Indeed, a recent study showed that Nrf2 expression is specifically repressed in maturing neurons [60]. However, astrocytic Nrf2 activation can be regulated by neuronal activity: increased neuronal synaptic activity and neurotransmitter release stimulates Nrf2 induction in co-cultured astrocytes but does not alter Nrf2 signaling when neurons are cultured alone, suggesting a critical interplay between neurons and astrocytes in the protective induction of Nrf2 signaling [61]. The results of the current study are entirely consistent with these investigations, whereby robust induction of Nrf2 was only evident in astrocytes, and all but absent in neurons including when cultured with astrocytes. These results indicate that astrocytes are more sensitive to PDTC-mediated induction of the Nrf2-ARE pathway than neurons. Furthermore, Nrf2 induction was more readily induced by PDTC in astrocytes cultured from adult mice as compared to neonatal mice. This is in line with our previous findings that adult astrocytes exhibit greater alterations in gene expression than neonatal astrocytes [62]. The PDTC-conferred enhancement of protective antioxidative mechanisms occurs specifically via activation of Nrf2 signaling, as evidenced by the absence of antioxidant induction in Nrf2-deficient astrocytes.

The mechanism via which PDTC induces the Nrf2 response likely involves multiple mechanisms, including decreased degradation, altered biometal homeostasis, and potential interplay with NF-κB. These will now be considered. Firstly, PDTC is a well-known inhibitor of the pro-inflammatory transcription factor NF-κB. However, there is much evidence for complex crosstalk between Nrf2 and NF-κB. Nrf2 can directly and indirectly suppress NF-κB signaling, whereas NF-κB can both amplify and diminish Nrf2 signaling [63, 64]. NF-κB can decrease Nrf2 binding to DNA by competing for co-activators and promoting a co-repressor [65]. Conversely, NF-κB can enhance Nrf2 signaling by promoting Nrf2 transcription [63]. It is unclear whether PDTC promotes Nrf2 signaling via inhibition of NF-κB or whether the well-known NF-κB inhibitory effects of PDTC are at least in part due to Nrf2 activation. Clearly, further studies are required to delineate the relationship between NF-κB inhibition and Nrf2 induction by PDTC.

The slight increase in cytosolic Nrf2 in addition to increased nuclear Nrf2 suggests that PDTC may stimulate de novo synthesis of Nrf2. Decreased cytosolic degradation is also likely to contribute to the observed elevations in Nrf2. Keap1 is a negative regulator of Nrf2 that promotes cytosolic degradation of Nrf2 [8]. PDTC treatment decreased Keap1 levels, likely reducing Nrf2 degradation and promoting Nrf2 accumulation. In addition, Nrf2 degradation may be reduced by GSK3β inhibition. GSK3β is a kinase that modulates activity of Nrf2-ARE signaling by promoting Keap1-independent Nrf2 degradation [66, 67]. Direct inhibition of GSK3β results in nuclear accumulation of Nrf2, indicating that GSK3β is a fundamental element of Nrf2-ARE regulation after oxidative injury [68, 69]. It is known that PDTC exerts its protective effects via reduction of activated GSK3β signaling in AD [41] and in neonatal hypoxia-ischemia [34]. In agreement with previous studies, we found that PDTC also dampens GSK3β activity in primary astrocytes. Direct inhibition of GSK3β also induced Nrf2 in the current study, although to a lesser extent than PDTC. Inhibition of kinases upstream of GSK3β including MAPK and PI3K/Akt failed to influence Nrf2 induction by PDTC, although this is likely due to redundancy within the kinase pathways. Taken together, it seems that GSK3β is implicated in PDTC-mediated Nrf2 induction. As the activity of GSK3β is known to be influenced by metals [70, 71] and PDTC is reported to bind metals and transport them inside cells, the observed effects could also be related to the metal-chelating property of PDTC [72, 73].

Copper is a trace element that is essential for cellular function. Disturbances in copper homeostasis are detrimental and lead to disease [74, 75]. We show here that a 2-week treatment of mice with PDTC results in an increased copper concentration in the brain. As copper is known to induce the ERK [76] and Akt pathways [77], we hypothesize that the PDTC-mediated increase in intracellular copper could trigger the phosphorylation of ERK and Akt, leading to reduced GSK3β activity (increased inhibitory phosphorylation) [78]. In support of our hypothesis, it has been shown that copper exposure induces nuclear accumulation of Nrf2 and induces protective Nrf2 signaling [79], which is blocked in Nrf2-deficient cells [80]. It is of interest to note that Keap1, the stress sensor and negative regulator of Nrf2, quantifies cellular stress by monitoring intracellular levels of metals such as zinc [81]. Therefore, it seems plausible that PDTC-mediated metal transport is responsible for the observed induction of the Nrf2 signaling in this study. Further studies are clearly required to thoroughly assess the interplay between metal homeostasis and Nrf2.

Our findings on the inhibition of GSK3β by PDTC are particularly interesting in the context of AD, where GSK3β not only phosphorylates tau protein [82] but also affects the expression of Aβ degrading enzymes [83]. The interplay between Aβ and Nrf2 is highlighted by our finding that treatment of cells with Aβ resulted in increased expression of the Nrf2 target gene HO-1 in astrocytes, in line with previous studies [84] and reported increases in HO-1 expression in the AD brain [85]. Furthermore, the increase in HO-1 expression, and that of Nrf2 target gene GCLM, was significantly augmented in astrocytes co-treated with PDTC and Aβ. It should be noted that although HO-1 expression can be regulated by other transcription factors besides Nrf2 [86, 87] and therefore making it possible that HO-1 induction may additionally involve other transcription factors, the induction of GCLM indicates that Nrf2 signaling is induced. These results indicate that while Aβ itself has the potential to slightly induce Nrf2 target gene expression, enhanced up-regulation of the protective pathway by PDTC is achieved in an elevated Aβ environment. These data suggest that an AD-related environment is especially favorable for effective induction of protective Nrf2 signaling by PDTC.

We observed a reduction of hippocampal lipid peroxidation with PDTC treatment in tissue homogenates in vivo, suggesting that a more antioxidative environment has been induced. That we detect elevated hydroperoxides in Nrf2-deficient mice supports the notion that Nrf2 is involved in the decreased lipid peroxidation induced by PDTC. The fact that we did not observe a significant induction of other Nrf2-ARE pathway markers in tissue homogenates in vivo is not completely unexpected given the in vitro data shown in this study: robust PDTC-mediated activation of Nrf2 signaling was only observed in astrocytes. While astrocytes have traditionally been viewed as the most abundant cell type in the brain, recent reports demonstrate that in the mouse brain, approximately 35 % of the cells are non-neuronal [88, 89]. This 35 % includes astrocytes, microglia, oligodendrocytes, and various precursor cells. The homogenized tissue samples used in this study for mRNA and protein expression analyses exclude the possibility of discerning the responses of specific cell types; hence, any astrocyte-specific effects will be masked by the presence of other cell types in the tissue homogenates. Indeed, our immunohistochemical analyses of brain showed HO-1 induction in only certain cell types in vivo, indicating that PDTC exerts cell-specific effects also in the brain. It should be noted that the current study did not assess the potential of PDTC to induce Nrf2-ARE signaling in microglial cells. It is therefore possible that the beneficial effects of PDTC on Nrf2 induction also occur in these cells.

Finally, strong in vitro activation of Nrf2 signaling was observed in the presence of Aβ. As the current study utilized only WT mice for assessing the effects of PDTC on Nrf2-ARE induction, it can be speculated that PDTC may also more strongly induce Nrf2-ARE signaling in vivo if Aβ is present. Given that the Nrf2 pathway is impaired in AD mice and in the brains of AD patients [12, 15], further studies are needed to assess the contribution of Aβ to PDTC-induced Nrf2 activation in vivo.

## Conclusions

In this study, we have identified a novel function of the metal chelator PDTC: the activation of Nrf2 signaling in astrocytes. Furthermore, activation of Nrf2 was augmented in the presence of Aβ, and PDTC decreased brain lipid peroxidation. These effects may contribute to the neuroprotection we and others have observed for PDTC in models of AD [41, 42]. The identification of safe, well-tolerated molecules that cross the blood-brain barrier to activate the Nrf2-ARE pathway is important, as these may have a valuable role in protecting the brain from oxidative stress associated with neurodegenerative diseases.

## Additional files

Additional file 1: Figure S1. Effect of PDTC treatment on viability of astrocytes and hippocampal neurons. (A) Primary cultures of neonatal astrocytes were treated with increasing concentrations of PDTC for 24 h, after which cell viability was measured by LDH release and (B) MTT reduction assays. (C) Hippocampal neuron survival following 24-h PDTC treatment was measured by manual counting of viable cells/all cells following Hoechst staining. Data are expressed as mean % of viable cells ± SD and normalized to control cells in (B) and (C). ***p* < 0.001 relative to corresponding control. Additional file 2: Figure S2. Absence of Nrf2 immunostaining in Nrf2^−/−^ astrocytes. Representative Nrf2 immunostaining images of primary Nrf2^−/−^ astrocytes treated with 100 μM PDTC for 4 h. DAPI staining was carried out to visualize nuclei. Scale bar = 40 μm. Additional file 3: Figure S3. Inhibition of kinases does not alter Nrf2 induction by PDTC. (A,E) Representative immunoblots with antibodies against Nrf2, HO-1, and GAPDH in primary neonatal astrocytes co-treated for 4 h with PDTC and (A) SL327 or PD198306 (MEK inhibitors), or (E) LY294002 (PI3K inhibitor) or FPA124 (Akt inhibitor). (B,F) Quantification of Nrf2 and (C,G) HO-1 protein expression after 4 h co-treatment of astrocytes with PDTC and indicated inhibitors. Data are expressed as fold change over control cells in absence of inhibitors, normalized to GAPDH ± SD. (D,H) Exported glutathione after 4 h PDTC co-treatment of neonatal astrocytes in presence of (D) SL327 or PD98059 (MEK inhibitors), or (H) LY294002 or PI828 (PI3K inhibitors), FPA124 or 10-DEBC (Akt inhibitors). Data expressed as nanomole glutathione released into the medium per well. Additional file 4: Figure S4. Effect of kinase inhibitors on viability of astrocytes. (A) Cell viability was measured by LDH release and (B) MTT reduction in primary neonatal astrocytes co-treated with PDTC and 10 μM indicated inhibitors for 4 h. LDH release expressed as mean percentage of initial total cellular LDH activity ± SD. MTT reduction expressed as mean percentage of viable cells ± SD normalized to control cells. **p* < 0.05 relative to corresponding control cells; ^a^ *p* < 0.05 relative to corresponding treatment without PDTC. Additional file 5: Figure S5. Expression of Nrf2-pathway related genes and proteins in mouse hippocampi following PDTC treatment. WT mice were treated with PDTC in the drinking water at a dose of 75 mg/kg/day for 2 weeks, or for 9 months at a dose of 20 mg/kg/day. (A) HO-1, (B) NQO1, and (C) Nrf2 mRNA expression levels were measured by quantitative RT-PCR in hippocampal samples and are presented as mean relative expression normalized to β-actin ± SD. (D) Representative immunoblots with antibodies against Nrf2, NQO1, and β-actin with hippocampal samples from mice treated 2 weeks with PDTC at a dose of 75 mg/kg/day. (E) Quantification of hippocampal Nrf2 protein levels after PDTC treatment of mice. Data are expressed as fold change over control cells normalized to β-actin ± SD. (F) Quantification of hippocampal NQO1 protein levels after PDTC treatment of mice. Data are expressed as fold change over control cells normalized to β-actin ± SD.

## Abbreviations

AD: Alzheimer’s disease
ARE: antioxidant response element
Aβ: amyloid-beta
CST: Cell Signaling Technology
GCL: glutamate-cysteine ligase
GCLC: glutamate-cysteine ligase catalytic subunit
GCLM: glutamate-cysteine ligase modifier subunit
GSH: glutathione
GSK3: glycogen synthase kinase 3
HO-1: heme oxygenase-1
LDH: lactate dehydrogenase
MEK: mitogen-activated protein kinase kinase
NF-κB: nuclear factor-κB
NQO1: NAD(P)H:quinone oxidoreductase-1
Nrf2: nuclear factor erythroid 2-related factor 2
PDTC: pyrrolidine dithiocarbamate
ROS: reactive oxygen species
WT: wild-type
γGC: gamma-glutamylcysteine
