# Supplementary figures and images for: Pyrrolidine dithiocarbamate activates the Nrf2 pathway in astrocytes
Source: J Neuroinflammation. 2016 Feb 26;13:49. doi: 10.1186/s12974-016-0515-9 (PMC4768425; doi:10.1186/s12974-016-0515-9)

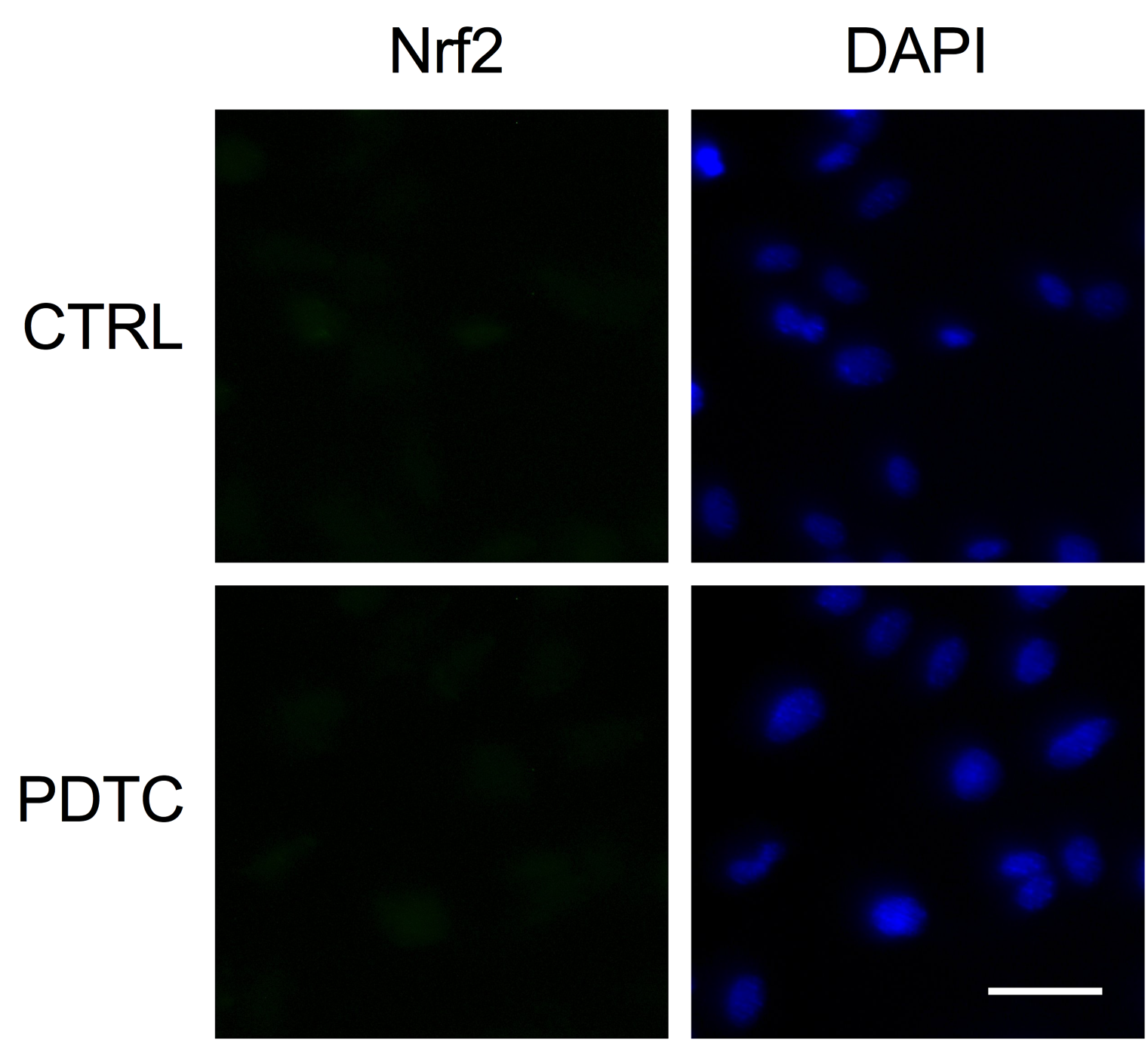

Supplement: Additional file 2: Figure S2. — Absence of Nrf2 immunostaining in Nrf2−/− astrocytes. Representative Nrf2 immunostaining images of primary Nrf2−/− astrocytes treated with 100 μM PDTC for 4 h. DAPI staining was carried out to visualize nuclei. Scale bar = 40 μm. [file 12974_2016_515_MOESM2_ESM.tiff]
